# Variable exposure to echocardiography core competencies when applying minimum recommended procedural numbers for cardiology fellows in training

**DOI:** 10.1186/s12947-022-00294-1

**Published:** 2022-09-20

**Authors:** Matthew J. Bierowski, Umer Qureshi, Shayann Ramedani, Simran Grewal, Ravi Shah, Robert Park, Brandon R. Peterson

**Affiliations:** 1 Department of Medicine, Penn State College of Medicine, Hershey, PA 17033 USA; 2 Department of Nuclear Cardiology, Massachusetts General Hospital, Harvard Medical School, Boston, MA 02115 USA; 3 Penn State Heart and Vascular Institute, Penn State College of Medicine, Box H047, 500 University Drive, Hershey, PA 17033 USA

**Keywords:** Cardiology fellowship, COCATS, Competency-based education

## Abstract

**Background:**

The American College of Cardiology Core Cardiovascular Training Statement (COCATS) defined echocardiography core competencies and set the minimum recommend number of echocardiograms to perform (150) and interpret (300) for independent practice in echocardiography (level 2 training). Fellows may lack exposure to key pathologies that are relatively infrequent, however, even when achieving an adequate number of studies performed and interpreted. We hypothesized that cardiology fellows would lack exposure to 1 or more cardiac pathologies related to core competencies in COCATS when performing and interpreting the minimum recommend number of studies for level 2 training.

**Methods:**

We retrospectively reviewed 11,250 reports from consecutive echocardiograms interpreted (7,500) and performed (3,750) by 25 cardiology fellows at a University tertiary referral hospital who graduated between 2015 and 2019. The first 300 echocardiograms interpreted and the first 150 echocardiograms performed by each fellow were included in the analysis. Echocardiography reports were reviewed for cardiac pathologies relating to core competencies defined in COCATS.

**Results:**

All 25 fellows lacked exposure to 1 or more cardiac pathologies related to echocardiography core competencies despite meeting COCATS minimum recommended numbers for echocardiograms performed and interpreted. Pathologies for which 1 or more fellows encountered 0 cases despite meeting the minimum recommended numbers for both echocardiograms performed and interpreted included: pericardial constriction (16/25 fellows), aortic dissection (15/25 fellows), pericardial tamponade (4/25 fellows), valvular mass/thrombus (2/25 fellows), prosthetic valve dysfunction (1/25 fellows), and cardiac chamber mass/thrombus (1/25 fellows).

**Conclusions:**

Cardiology fellows who completed the minimum recommend number of echocardiograms performed and interpreted for COCATS level 2 training frequently lacked exposure to cardiac pathologies, even in a University tertiary referral hospital setting. These data suggest that fellowship programs should monitor pathology case counts for each fellow in training, in addition to the minimum recommend number of echocardiograms defined by COCATS, to ensure competency for independent practice in echocardiography.

## Background

Standards for cardiology fellowship training in echocardiography were defined in the American College of Cardiology Core Cardiovascular Training Statement (COCATS 4) from 2015 [1, 2]. These standards, released by the American College of Cardiology and endorsed by the American Society of Echocardiography, provide guidance regarding core competencies to achieve during the course of echocardiography training. Recommended procedural numbers for independent practice in echocardiography (level 2 certification) include a minimum of 150 echocardiograms performed and a minimum of 300 echocardiograms interpreted during cardiology fellowship training. Beyond recommended procedural numbers, core competencies are also outlined in the training statement and span 6 domains as recommended by the Accreditation Council for Graduate Medical Education [3]. Competencies within the “Medical Knowledge” domain for level 2 certification in echocardiography include: quantifying ventricular size and function, evaluating for native and prosthetic valve diseases, recognizing characteristics of pericardial disease, assessing right heart disease and pulmonary hypertension, identifying cardiac masses such as thrombus or vegetation, among others [2]. The minimum recommended numbers for level 2 certification are meant to ensure a broad exposure to cardiac pathologies and core competencies in echocardiography required for independent practice. The variable incidence of different cardiac pathologies, however, could impact whether fellows have adequate exposure to all key cardiac pathologies related to core competencies within the minimum recommended numbers to perform and interpret. Exposure to these various pathologies and core competencies may differ from program to program or even from fellow to fellow within the same training program. For example, uncommon pathologies may be encountered more frequently by cardiology fellows training in a University tertiary referral institution than in a community-based hospital. Given the variable incidence of cardiac pathologies, we hypothesized that cardiology fellows would lack exposure to 1 or more cardiac pathologies related to core competencies in COCATS when performing and interpreting the minimum recommend number of studies for level 2 training.

## Methods

We retrospectively reviewed echocardiography reports from 25 consecutive cardiology fellows in training at Penn State Hershey Medical Center, a 637 bed tertiary care academic medical center. We reviewed echocardiography reports from cardiology fellows at our institution who completed training from June 2015 through June 2019 (5 fellows per year). We extracted echocardiography reports from the first 300 echocardiograms interpreted and the first 150 echocardiograms performed for each fellow, without exclusions, corresponding to the minimum recommended numbers for level 2 certification in COCATS for independent practice in echocardiography. In total, we reviewed and analyzed data from 3,750 reports of echocardiograms performed and 7,500 reports of echocardiograms interpreted for this study.

We extracted findings of cardiac pathologies from echocardiography reports corresponding to core competencies described in COCATS (see Table 1). For sample pathologies of valve disease, we focused on stenosis and regurgitation of the aortic valve and mitral valve. We included mitral stenosis and aortic stenosis when either was reported to be at least mild in severity, and we included mitral regurgitation and aortic regurgitation when either was reported to be greater than mild in severity. Prosthetic valve dysfunction was defined as reporting of significant prosthetic stenosis, prosthetic valve regurgitation (greater than mild), or patient-prosthetic mismatch. Echocardiograms labeled with either Current Procedural Terminology (CPT) 93,303 or 93,304 were categorized as presence of adult congenital heart disease for this study, and included both simple congenital heart disease (eg atrial septal defect, ventricular septal defect, bicuspid aortic valve, pulmonic stenosis) and more complex congenital heart disease (Tetralogy of Fallot, transposition of the great arteries, single ventricle defects, etc.).

**Table 1:** Medical Knowledge core competencies in echocardiography according to COCATS and corresponding cardiac pathologies extracted from echocardiography reports

Core Competencies	Cardiac Pathology
“Know the techniques to quantify cardiac chamber sizes and evaluate left and right ventricular systolic and diastolic function and hemodynamics.” “Know the characteristic findings of cardiomyopathies”	Left ventricular dilation Left ventricular systolic dysfunction Left ventricular wall motion abnormalities
“Know the techniques to assess pulmonary artery pressure and diseases of the right heart.”	Right ventricular dilation Right ventricular systolic dysfunction Pulmonary hypertension
“Know the echocardiographic findings of pericardial disease, pericardial effusion, and pericardial constriction.”	Pericardial constriction Pericardial effusion Pericardial tamponade
“Know the use of echocardiographic and Doppler data to evaluate native and prosthetic valve function and diseases.”	Aortic stenosis Aortic regurgitation Mitral stenosis Mitral regurgitation Prosthetic valve dysfunction (eg stenosis, regurgitation, or patient-prosthetic mismatch)
“Know the techniques to evaluate diseases of the aorta.”	Aortic dilation Aortic dissection
“Know the characteristic findings of basic adult congenital heart disease.”	Presence of adult congenital heart disease
“Know the techniques to evaluate cardiac masses and suspected endocarditis.” “Know the indications for, and the echocardiographic findings in, patients with known or suspected cardioembolic events.”	Valvular mass/thrombus Cardiac chamber mass/thrombus

For context in terms of cardiac surgery volume at our institution, from 2015 through 2019, there were 2,066 cardiac surgeries performed at our institution, including 896 coronary artery bypass graft (CABG) operations, 245 surgical aortic valve replacements (not including transcatheter aortic valve replacements), 140 combination CABG plus AVR, 222 mitral valve repair or replacement procedures (either alone or combined with CABG or other valve repair/replacement), and 563 additional cardiac surgeries beyond those categorized above.

Any and all pathologies that were reported for each echocardiography study as defined in Table 1 were recorded from the first 300 echocardiograms interpreted and the first 150 echocardiograms performed by each fellow.

Statistical analysis was performed using Excel (Microsoft Office Professional Plus 2019). For statistical analyses, we used paired, two-tailed T-test to compare the mean values of pathologies encountered per cardiology fellow. Paired t-testing was used for analysis because each pathology analyzed was recorded and reported by the same group of 25 cardiology fellows.. A *p*-value of less than 0.05 was considered significant. We calculated the median and interquartile range for the number of instances a pathology was encountered by each cardiology fellow to demonstrate variance in exposure to pathologies among all cardiology fellows.

Absolute deficiencies and relative deficiencies in exposure to core competencies were determined for each cardiology fellow. We defined an absolute deficiency as 0 exposures to a pathology within the first 300 echocardiograms interpreted, and as 0 exposures to a pathology within the first 150 echocardiograms performed. We defined a relative deficiency as at least 1, but less than 10, cases encountered of a pathology within the first 300 echocardiograms interpreted or within the first 150 echocardiograms performed.

## Results

There were a total of 12,911 pathologies reported in the echocardiograms interpreted and 6,917 pathologies reported in the echocardiograms performed. Table 2 shows the frequencies of each pathology among echocardiograms interpreted and performed. On review of the patient population for which echocardiograms were performed or interpreted, the average patient age was 63.6 with a range from 16 to 106 years old. 53.9% of patients who underwent echocardiography during that same time period were male.

**Table 2:** Frequency of cardiac pathologies according to core competency

Core Competency	Cardiac Pathology	Echocardiograms Interpreted n (%)	Echocardiograms Performed n (%)
Left ventricular function/disease	Left ventricular dilation	1236 (16.5%)	710 (18.9%)
	Left ventricular systolic dysfunction	1754 (23.4%)	1042 (27.8%)
	Left ventricular wall motion abnormalities	2047 (27.3%)	1203 (32%)
Right ventricular function/disease	Right ventricular dilation	1735 (23.1%)	984 (26.2%)
	Right ventricular systolic dysfunction	1158 (15.4%)	714 (19%)
	Pulmonary hypertension	1020 (13.6%)	545 (14.5%)
Pericardial disease	Pericardial constriction	8 (0.1%)	3 (0.1%)
	Pericardial effusion	370 (4.9%)	238 (6.3%)
	Pericardial tamponade	43 (0.6%)	37 (1%)
Valvular disease	Aortic stenosis	770 (10.3%)	293 (7.8%)
	Aortic regurgitation	322 (4.3%)	121 (3.2%)
	Mitral stenosis	158 (2.1%)	42 (1.1%)
	Mitral regurgitation	956 (12.8%)	425 (11.3%)
	Prosthetic valve dysfunction	69 (0.9%)	31 (0.8%)
Aortic disease	Aortic dilation	629 (8.4%)	262 (7%)
	Aortic dissection	8 (0.1%)	3 (0.1%)
Congenital heart disease	Adult congenital heart disease	175 (2.3%)	47 (1.3%)
Cardiac masses	Valvular mass/thrombus	45 (0.6%)	39 (1%)
	Cardiac mass/thrombus	102 (1.4%)	50 (1.3%)

**Fig. 1:**
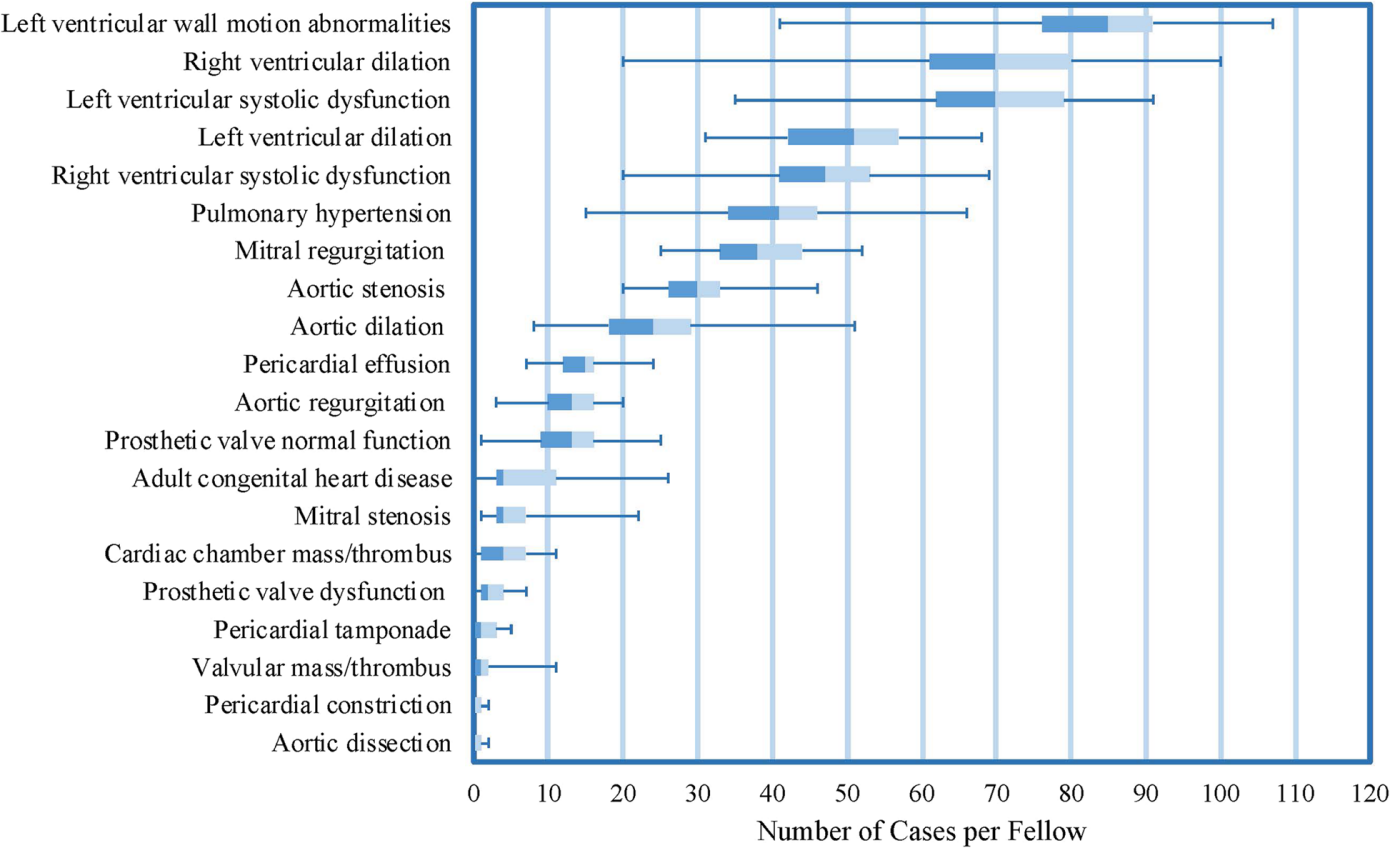
Box and whisker plot displaying pathologies encountered per fellow among the first 300 echocardiograms interpreted, ordered from most frequent to least frequent based on median value. Median values are displayed along with interquartile range (box) and minimum and maximum values (whiskers)

**Fig. 2:**
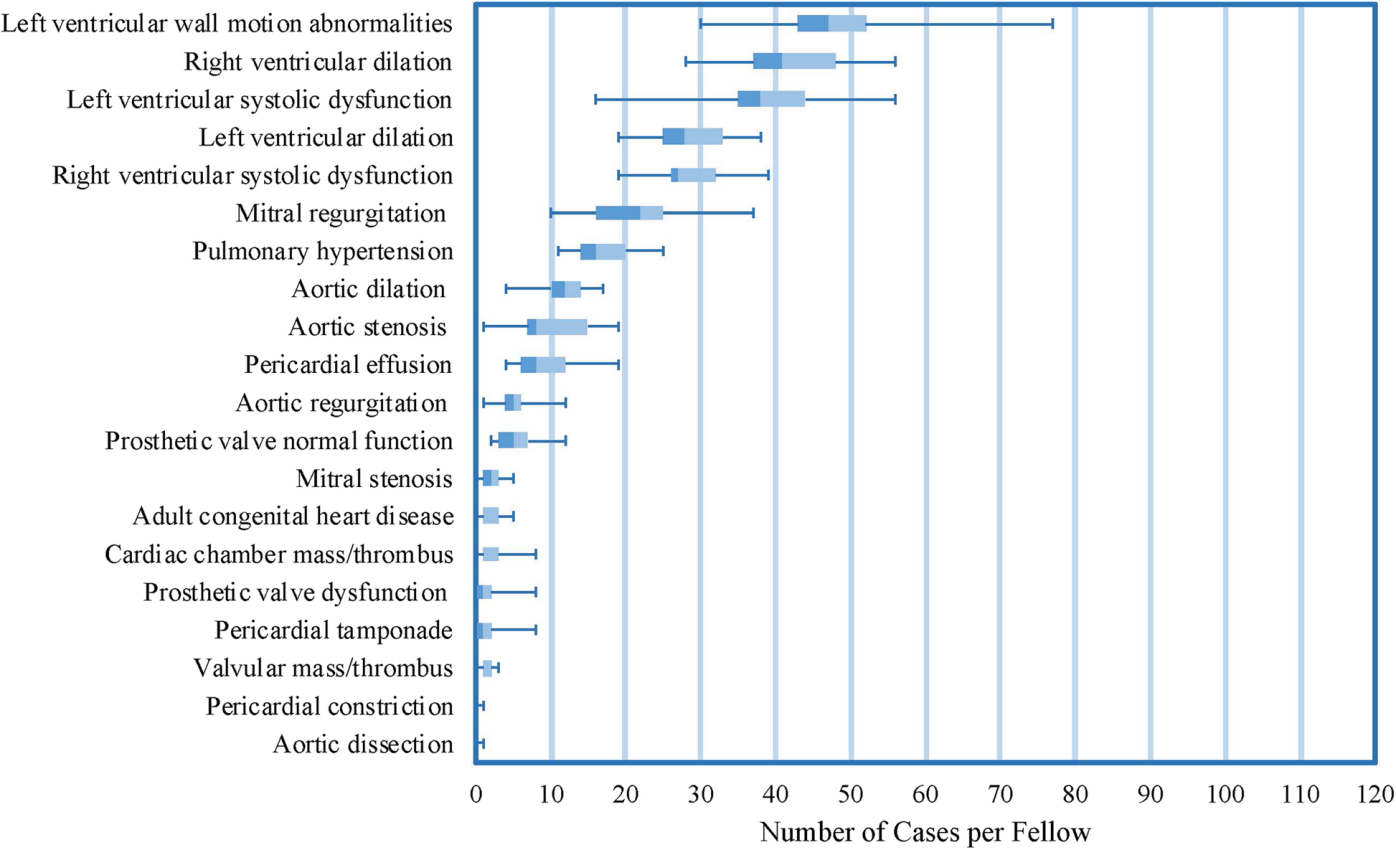
Box and whisker plot displaying pathologies encountered per fellow among the first 150 echocardiograms performed, ordered from most frequent to least frequent based on median value. Median values are displayed along with interquartile range (box) and minimum and maximum values (whiskers)

Left ventricular wall motion abnormalities was the most frequently reported pathology for both echocardiograms interpreted (mean 82 cases per fellow, standard deviation 17) and echocardiograms performed (mean 48 cases per fellow, standard deviation 12). Among echocardiograms interpreted, in comparison to left ventricular wall motion abnormalities, each other pathology was significantly less frequent (*p* < 0.001). Among echocardiograms performed, in comparison to left ventricular wall motion abnormalities, all other pathologies were significantly less frequent (*p* = 0.001 compared to left ventricular systolic dysfunction and *p* < 0.001 for all other comparisons). Aortic dissection and pericardial constriction were the least frequent pathologies among both echocardiograms interpreted and performed. Figures 1 and 2 display the pathologies encountered per fellow from most to least frequent among echocardiograms interpreted and echocardiograms performed, respectively.

**Fig. 3:**
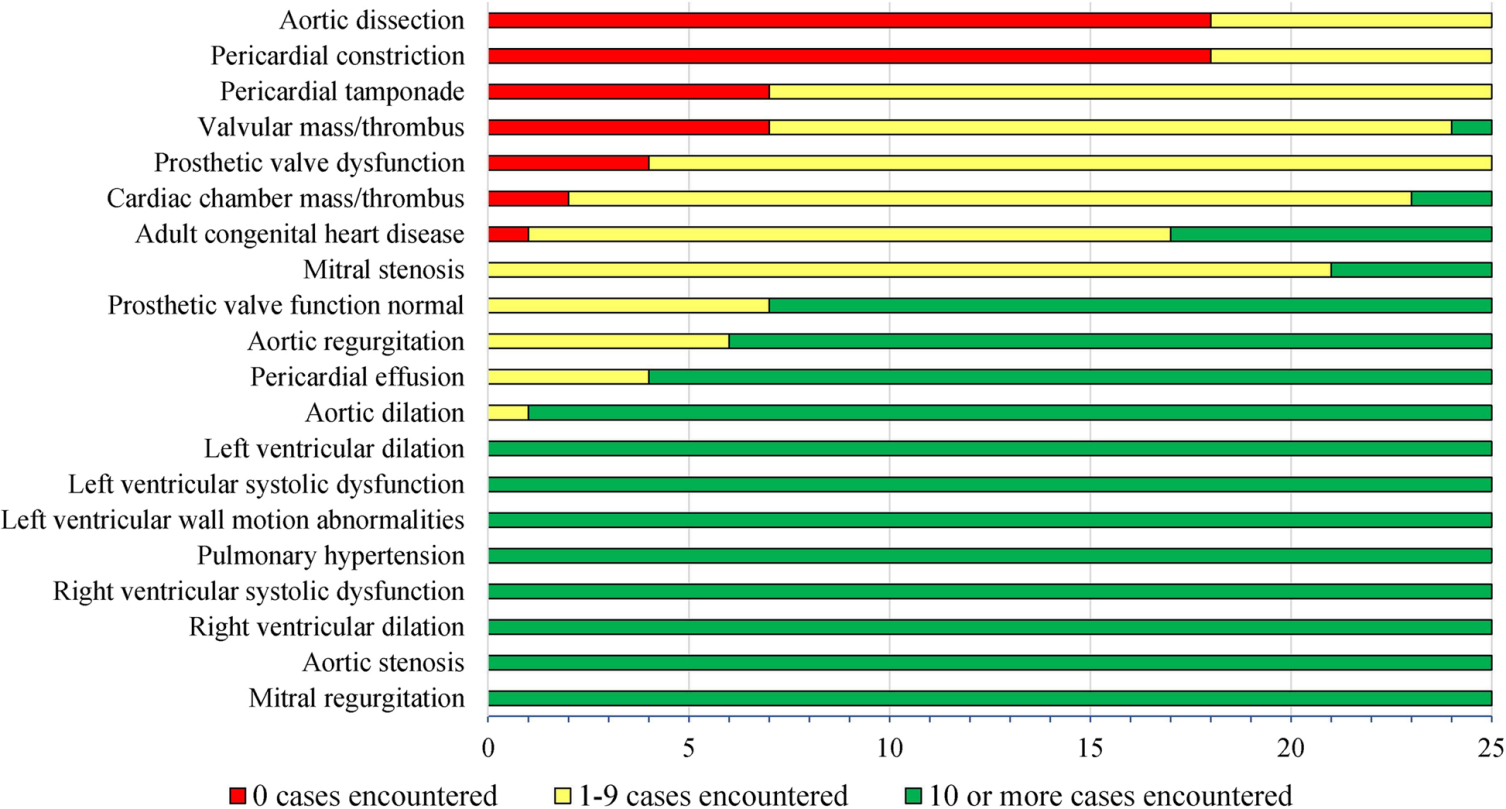
Color-coded bar graph displaying the number of fellows (out of 25 total) with deficiencies in encountering cardiac pathologies related to core competencies among the first 300 echocardiograms interpreted, with green representing no deficiency (10 or more cases encountered), yellow representing relative deficiency (1–9 cases encountered) and red representing absolute deficiency (0 cases encountered). Pathologies are sorted from most to least deficiencies encountered

**Fig. 4:**
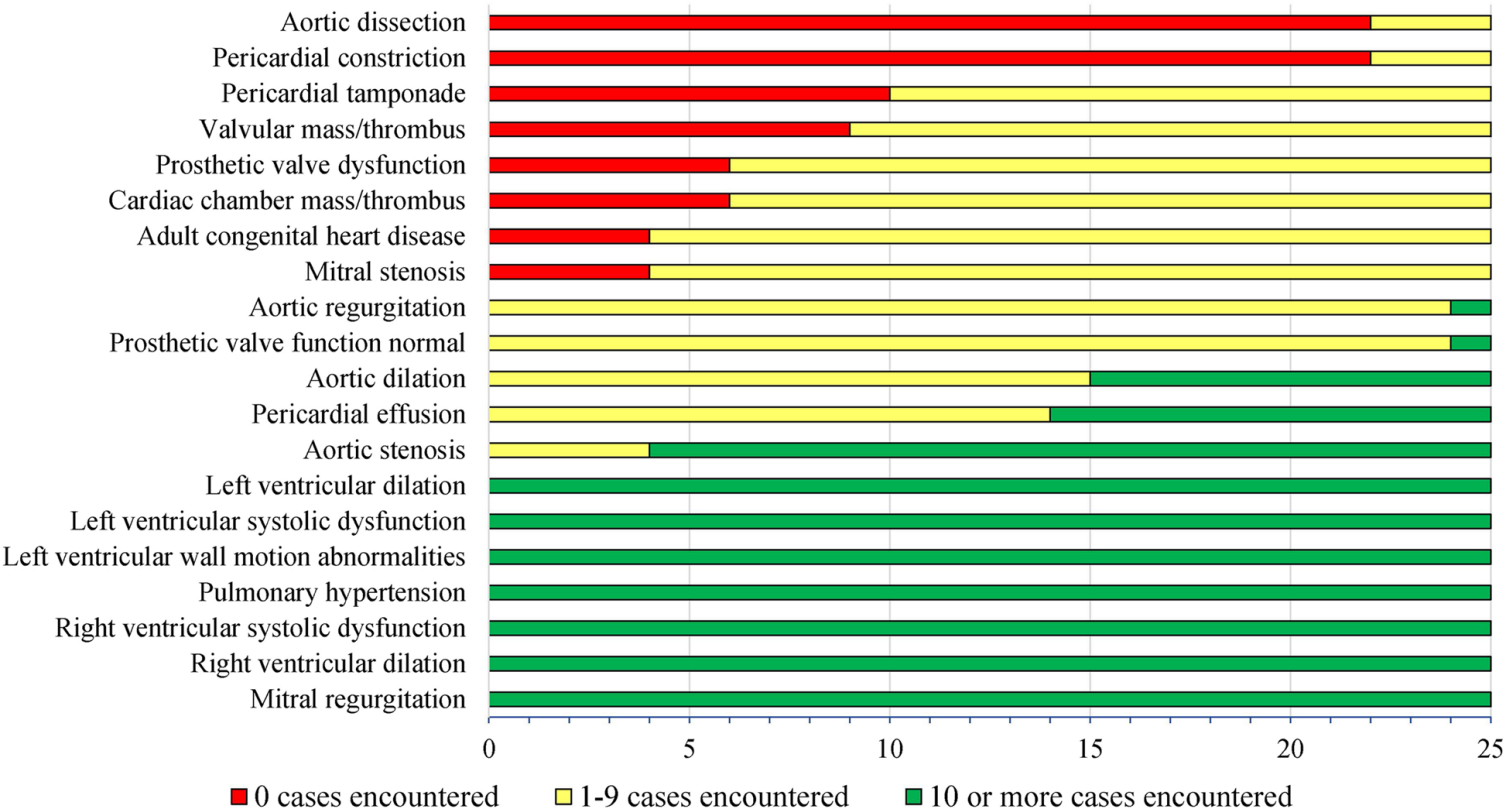
Color-coded graph displaying the number of fellows (out of 25 total) with deficiencies in encountering cardiac pathologies related to core competencies among the first 150 echocardiograms performed, with green representing no deficiency (10 or more cases encountered), yellow representing relative deficiency (1–9 cases encountered) and red representing absolute deficiency (0 cases encountered). Pathologies are sorted from most to least deficiencies encountered

All 25 fellows had absolute deficiencies in 1 or more areas of pathology relating to the core competencies in the first 150 echocardiograms performed, and in 1 or more areas of pathology in the first 300 echocardiograms interpreted. Pathologies for which cardiology fellows encountered 0 cases despite meeting the recommended numbers for both echocardiograms performed and interpreted included: pericardial constriction (16/25 fellows), aortic dissection (15/25 fellows), pericardial tamponade (4/25 fellows), valvular mass/thrombus (2/25 fellows), prosthetic valve dysfunction (1/25 fellows), and cardiac chamber mass/thrombus (1/25 fellows). Figure 3 (echocardiograms interpreted) and Fig. 4 (echocardiograms performed) highlight the number of fellows with deficiencies in encountering pathologies.

## Discussion

The American College of Cardiology Core Cardiovascular Training Statement (COCATS) defines echocardiography core competencies to achieve in fellowship training, as well as the minimum recommended number of echocardiograms to perform (150) and interpret (300) for independent practice in echocardiography. Our study demonstrated that cardiology fellows lacked exposure to a number of cardiac pathologies related to core competencies in their echocardiography training despite meeting the minimum recommended numbers for level 2 training by COCATS. The relative infrequency of certain pathologies among the core competencies defined in COCATS suggests that fellowship programs should consider monitoring pathology case counts for each fellow, beyond overall number of studies interpreted, to ensure adequate exposure during the course of fellowship training.

Gaps in exposure to cardiac pathologies during echocardiography training may lead to lack of proficiency in interpreting pathology during clinical practice. To assess fellow proficiency in echocardiography, Nair et al. investigated third year cardiology fellows who had performed a mean of 261 echocardiograms and interpreted a mean of 353 echocardiograms during cardiology training (both exceeding level 2 COCATS standards) and found multiple areas in which 50% or more of fellows failed to achieve a passing score on a dedicated echocardiography interpretation observed structured clinical examination (OSCE). Clinical scenarios where fellows failed to demonstrate proficiency in interpretation included ascending aortic aneurysm complicated with aortic regurgitation (44% pass rate) and interpretation of bioprosthetic valve dysfunction (11% pass rate) [4]. Only a weak correlation was found between the number of echocardiograms interpreted and interpretations scores on the OSCE (*r* = 0.33) [4], suggesting that total procedural numbers may be inadequate to reflect proficiency. Our study expands on this prior research by demonstrating that multiple cardiac pathologies described in COCATS are encountered much less frequently during echocardiography training; this may help to explain fellows’ lack of proficiency in interpreting pathologies such as prosthetic valve dysfunction despite exceeding minimum recommend numbers.

We found that the lower numeric requirement of echocardiograms to perform (at least 150) compared to echocardiograms to interpret (at least 300) resulted in an increase in absolute and relative deficiencies to cardiac pathologies among fellows when comparing echocardiograms performed to echocardiograms interpreted. This discrepancy may disproportionately affect cardiology fellows’ ability to identify and evaluate pathology when performing echocardiography. A prior survey of graduating cardiology fellows regarding self-perceived echocardiography competency found that a greater percentage of fellows perceived themselves to be “highly” or “extremely highly” proficient at interpreting echocardiography (78%) as compared to performing echocardiography (54%) [5]. Our study may explain these findings in part by demonstrating that a reduced exposure to cardiac pathologies among echocardiograms performed, due to lower numeric training requirements, may contribute to this self-perception of inadequate competency in performing echocardiography.

The implications of our study extend beyond fellow competency in echocardiography. Training statements as part of COCATS 4 cover a wide variety of skills and study interpretation, including competencies in cardiac MRI, nuclear cardiology, and cardiovascular computed tomographic imaging, among others. In each case, expected competencies and milestones are outlined for proficiency, along with recommended minimum procedural numbers and time in training for each area. Chow et al. found that in the area of nuclear cardiology, fellows demonstrated variables rates of achieving competency in interpretation based on agreement with attending final interpretation, and on average cardiology fellows required higher numbers of procedures to achieve competency beyond the minimum number of studies recommended in COCATS [6]. Fellowship programs should consider adopting a strategy of monitoring pathology case counts, encountered through a combination of study interpretation, case conferences, and didactics, to ensure adequate exposure to each area of core competency.

Cardiology fellowship programs may use a variety of approaches to broaden fellows’ exposure to echocardiography pathologies when deficiencies in pathology case counts are identified. Strategies may include lecture series, case conferences to review key cardiac pathologies, and/or case logs to document the variety of pathologies encountered. Attending physicians may consider archiving cases with uncommon or rare yet significant pathologies for fellows to review to ensure adequate exposure to key pathologies. Direct observations, as well as structured in-training assessments such as OSCEs, may also enable fellowship programs to identify deficiencies and enable targeted feedback. A study by Nielsen et al. described use of an OSCE for assessing technical proficiency in performing transthoracic echocardiography in patients with normal cardiac function as well as with aortic stenosis and with mitral regurgitation, which assessment tool fellowship programs may find useful as a model for assessing competency in performing echocardiography [7]. Fellowship clinical competency committees may consider different methods for providing appropriate assessment and feedback during echocardiography training [8].

Simulated echocardiography, where available, may offer another modality for supplementing core competencies in echocardiography education. Use of echocardiography simulation has been demonstrated to be an effective tool for teaching and assessment of competency in transthoracic [9, 10] and transesophageal [11–14] echocardiography. Current software is now capable of simulating not only normal cardiac function but also valve dysfunction, abnormal wall motion, cardiac tamponade, and aortic dissection, among others, allowing fellows to have standardized modules for assessment and practice, particularly in areas of potential deficiency [15]. Our data suggest that use of such modules could be useful given the rarity of certain cardiac pathologies included in COCATS. Additionally, for programs without access to simulation mannequins, the use of online echocardiography simulation software exists which may be used to supplement learning [16, 17], with limited studies suggesting that use of online simulation resources may also be beneficial for echocardiography training [18, 19].

A number of important limitations should be considered in reviewing our results. As a retrospective study, our data were extracted from echocardiography reports. Exposure to certain echocardiography pathologies may have been under-counted if these pathologies were not specified in the final echocardiography report. Our study was a review of fellows within a single training program; specific areas of deficiency in echocardiography exposure are likely to vary at other institutions. Our institution has a dedicated adult congenital heart disease program, along with cardiac surgery and structural heart programs. Cardiology fellowship programs at smaller centers without these programs may find that fellows encounter less instances of certain cardiac pathologies, such as prosthetic valve dysfunction or congenital heart disease, for example.

All fellows at our institution exceeded to some degree the minimum recommended numbers in training, and therefore many may have encountered additional cardiac pathologies when accounting for their total numbers performed and interpreted. Additionally, fellows may gain exposure to key cardiac pathologies in other rotations even when they are not involved as the primary interpreter or performer of the echocardiogram, such as exposure to pericardial tamponade and constriction as the fellow in the cardiac catheterization laboratory, or to aortic dissection as the fellow rounding in the cardiovascular intensive care unit. Systems developed by fellowship programs to track pathology exposure across training will help to elucidate areas of deficiency that may not be readily apparent from individual fellow case counts of studies performed and interpreted. Further research is also needed to determine whether gaps in exposure to cardiac pathologies during fellowship training lead to meaningful impacts in study interpretation and patient outcomes during clinical practice.

## Conclusions

In summary, we found that cardiology fellows experience variable exposure, and occasionally lack of exposure, to cardiac pathologies even with meeting standards for minimum recommended numbers in training. This occurs in part due to the relative infrequency of several cardiac pathologies described in COCATS. This study highlights the importance of avoiding use of the minimum recommended numbers of studies to perform or interpret as a surrogate of competency or adequate exposure to pathology. We recommend that cardiology fellowship programs monitor pathology-specific case counts, beyond overall case counts of studies performed and interpreted, to inform supplemental didactics as needed in the curriculum and to ensure broad exposure to pathology for independent practice in echocardiography.

## Data Availability

The datasets used for this study are available from the corresponding author on reasonable request.

## Abbreviations

COCATS: Core Cardiovascular Training Statement
CPT: Current Procedural Terminology
OSCE: Observed Structural Clinical Examination

## References

[1] Halperin JL, Williams ES, Fuster V, Fuster V, Halperin JL, Williams ES (2015). ACC 2015 Core Cardiovascular Training Statement (COCATS 4) (Revision of COCATS 3). J Am Coll Cardiol.

[2] Ryan T, Berlacher K, Lindner JR, Mankad SV, Rose GA, Wang A (2015). COCATS 4 Task Force 5: Training in Echocardiography. J Am Coll Cardiol.

[3] Batalden P, Leach D, Swing S, Dreyfus H, Dreyfus S (2002). General competencies and accreditation in graduate medical education. Health Aff (Millwood).

[4] Nair P, Siu SC, Sloggett CE, Biclar L, Sidhu RS, Yu EHC (2006). The assessment of technical and interpretative proficiency in echocardiography. J Am Soc Echocardiogr.

[5] Yu EHC, Nair P, Sibbald MG, Lee DS, Dorian P (2015). Can diagnostic and procedural skills required to practice cardiology as a specialist be mastered in 3 years?. Can J Cardiol.

[6] Chow BJW, Alenazy A, Small G, Crean A, Yam Y, Beanlands RS (2019). competency-based medical education: do the cardiac imaging training guidelines have it right?. J Am Coll Cardiol Img.

[7] Nielsen DG, Gotzsche O, Eika B (2013). Objective structured assessment of technical competence in transthoracic echocardiography: a validity study in a standardised setting. BMC Med Educ.

[8] Ryan T, Wiegers SE (2019). Who Is a competent echocardiographer?. J Am Soc Echocardiogr.

[9] Neelankavil J, Howard-Quijano K, Hsieh TC, Ramsingh D, Scovotti JC, Chua JH (2012). Transthoracic echocardiography simulation is an efficient method to train anesthesiologists in basic transthoracic echocardiography skills. Anesth Analg.

[10] Montealegre-Gallegos M, Mahmood F, Kim H, Bergman R, Mitchell JD, Bose R (2016). Imaging skills for transthoracic echocardiography in cardiology fellows: the value of motion metrics. Ann Card Anaesth.

[11] Bick JS, DeMaria SJ, Kennedy JD, Schwartz AD, Weiner MM, Levine AI (2013). Comparison of expert and novice performance of a simulated transesophageal echocardiography examination. Simulation in Healthcare.

[12] Damp J, Anthony R, Davidson MA, Mendes L (2013). Effects of transesophageal echocardiography simulator training on learning and performance in cardiovascular medicine fellows. J Am Soc Echocardiogr.

[13] Matyal R, Montealegre-Gallegos M, Mitchell JD, Kim H, Bergman R, Hawthorne KM (2015). Manual skill acquisition during transesophageal echocardiography simulator training of cardiology fellows: a kinematic assessment. J Cardiothorac Vasc Anesth.

[14] Ferrero NA, Bortsov AV, Arora H, Martinelli SM, Kolarczyk LM, Teeter EC (2014). Simulator training enhances resident performance in transesophageal echocardiography. Anesthesiology.

[15] HeartWorks Pathology | Echocardiographic Evaluation | TEE & TTE. Intelligent Ultrasound. https://www.intelligentultrasound.com/heartworks/pathology/. Accessed 11 May 2022

[16] Inc K. Echocardiography training | Echo Test and Teach | KeLabs. https://www.kelabs.com/products/echo-test-teach. Accessed 11 May 2022

[17] Focused Cardiac Ultrasound Transthoracic Echocardiography TTE PIE Toronto FOCUS FATE Standard Views Tamponade Hypovolemia Ventricular dysfunction. http://pie.med.utoronto.ca/TTE/. Accessed 11 May 2022

[18] Vegas A, Meineri M, Jerath A, Corrin M, Silversides C, Tait G (2013). Impact of online transesophageal echocardiographic simulation on learning to navigate the 20 standard views. J Cardiothorac Vasc Anesth.

[19] Sharma V, Chamos C, Valencia O, Meineri M, Fletcher SN (2013). The impact of internet and simulation-based training on transoesophageal echocardiography learning in anaesthetic trainees: a prospective randomised study. Anaesthesia.

